# Fish–parasite interaction networks reveal latitudinal and taxonomic trends in the structure of host–parasite associations

**DOI:** 10.1017/S0031182022000944

**Published:** 2022-12

**Authors:** Robert Poulin, Cameron McDougall

**Affiliations:** Department of Zoology, University of Otago, P.O. Box 56, Dunedin 9054, New Zealand

**Keywords:** Bipartite network, coevolution, connectance, modularity, nestedness, taxonomy

## Abstract

In recent years, treating host–parasite associations as bipartite interaction networks has proven a powerful tool to identify structural patterns and their likely causes in communities of fish and their parasites. Network analysis allows for both community-level properties to be computed and investigated, and species-level roles to be determined. Here, using data from 31 host–parasite interaction networks from local fish communities around the world, we test for latitudinal trends at whole-network level, and taxonomic patterns at individual parasite species level. We found that while controlling for network size (number of species per network), network modularity, or the tendency for the network to be subdivided into groups of species that interact mostly with each other, decreased with increasing latitude. This suggests that tropical fish–parasite networks may be more stable than those from temperate regions in the event of community perturbations, such as species extinction. At the species level, after accounting for the effect of host specificity, we observed no difference in the centrality of parasite species within networks between parasites with different transmission modes. However, species in some taxa, namely branchiurans, acanthocephalans and larval trematodes, generally had higher centrality values than other parasite taxa. Because species with a central position often serve as module connectors, these 3 taxa may play a key role in whole-network cohesion. Our results highlight the usefulness of network analysis to reveal the aspects of fish–parasite community interactions that would otherwise remain hidden and advance our understanding of their evolution.

## Graphical abstract

**Figure:**
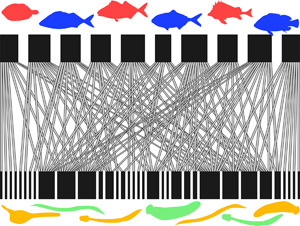

## Introduction

Communities can be loosely defined as a set of locally co-occurring species that can potentially interact. Several years ago, the study of these coexisting species, or community ecology, was deemed to be a mess of contingencies, with each system apparently following local rules and no way of predicting with any confidence what assembly and interaction rules a new, previously unstudied community would follow (Lawton, 1999). The lack of universal laws or predictable patterns seemed like an insurmountable obstacle towards understanding how species interact and coexist over time. Since then, however, much theoretical progress has been achieved to explain the various structuring forces acting to shape natural communities and determine their diversity and stability (Morin, 2011; Vellend, 2017; Leibold and Chase, 2018). Similarly, the community ecology of host–parasite interactions has also long sought to identify general underlying patterns and associated processes. On the scale of parasite species coexisting within the same host individual or the same host species, predictable gradients in parasite diversity or in the importance of interspecific interactions among parasites have long been known to exist (Esch *et al.*, 1990). However, at the larger scale of the entire host community including all their unique and shared parasites, generalizations have proven to be more elusive (Poulin, 2007), hindering progress in our understanding of host–parasite coevolution and parasite-mediated maintenance of biodiversity.

The use of network analysis has greatly remedied this, by providing a holistic tool for the study of host–parasite interactions within local communities (Poulin, 2010; Runghen *et al.*, 2021). Network analysis considers hosts and parasites as interconnected entities, thus capturing not only all species in a system, but also the interactions themselves, or links, between species. By treating hosts and parasites as 2 mutually interacting sets of species in a bipartite network, one can use a range of whole-network metrics to explore various structural aspects of the network, as well as species-level metrics to evaluate the role of individual species within the network.

At the whole-network level, the 3 most widely used and informative metrics are connectance, nestedness and modularity (Delmas *et al.*, 2019). Connectance, which is simply the proportion of all possible links that are realized, may be an important determinant of the stability of the network and its resilience to species loss (Delmas *et al.*, 2019). Nestedness provides a measure of the heterogeneous distributions of links among species (Bascompte *et al.*, 2003). In a highly nested network, specialist parasites infect a subset of the host species infected by generalist parasites, whereas host species with few parasites harbour parasite species that form subsets of those infecting hosts with richer parasite faunas. Finally, modularity measures the extent to which the network is divided into groups of species, or modules, having many interactions among themselves but few interactions with the members of other modules (Delmas *et al.*, 2019). Highly modular networks may indicate the existence of several distinct host–parasite coevolutionary units within the broader community. Nestedness and modularity are not totally independent of each other (Fortuna *et al.* 2010), and they both tend to covary with connectance (Delmas *et al.*, 2019), however, they each capture different aspects of network architecture.

At the level of individual species, several metrics measure the importance, position or role of particular species within the network, as a way of quantifying how each species influences the community by maintaining its cohesion and connecting other species (Delmas *et al.*, 2019). Among the most widely used are centrality measures. For example, betweenness centrality measures the proportion of times a species serves as a bridge on the shortest path connecting all other pairs of species within the network (Martín González *et al.*, 2010; Newman, 2018). Closeness centrality, in contrast, measures the average proximity of a species to all other species in the network. Several other approaches exist to quantify the most influential species within a network (e.g. Salavaty *et al.*, 2020), each considering slightly different aspects of network topology to derive a measure of species importance.

Fish communities and the parasites they harbour have been the subject of several network analyses (e.g. Bellay *et al.*, 2013, 2015). Many of these earlier studies consider only 1 or a few networks. Furthermore, the network-level or species-level metrics they use are based on different algorithms or computed with different criteria, and are therefore not easily comparable (Pellissier *et al.*, 2018; Xing and Fayle, 2021). Yet, a synthetic look at these fish–parasite networks can shed light on several key drivers of parasite community structure. For instance, at the whole-network level, do fundamental network properties such as connectance, nestedness and modulary vary along a latitudinal gradient? Given the generally higher species richness in tropical ecosystems (Willig *et al.*, 2003) and the tendency for consumers to be more specialized at low latitudes (Vázquez and Stevens, 2004; Krasnov *et al.*, 2008), we might expect variation in fish–parasite network properties as a function of latitude (see Guilhaumon *et al.*, 2012). At the species level, does the taxonomic affiliation of given parasite species, or their basic traits such as mode of transmission, determine their position within the network, measured as their centrality? Results from Bellay *et al.* (2013, 2015) suggest they might, as does an analysis of parasite species roles in whole food webs (Poulin *et al.*, 2013).

Here, following earlier studies by Bellay *et al.* (2013, 2015), we assembled a dataset comprising all publicly available host–parasite bipartite interaction networks involving fish and metazoan parasites, and subjected them to standardized analyses in order to obtain comparative data. Specifically, we address the following simple and basic questions: (i) while controlling for network size, do connectance, nestedness and modularity of fish–parasite networks vary with latitude? and (ii) do taxonomy and transmission mode explain the centrality of parasite species within fish–parasite networks? Our study illustrates the power of network analysis to reveal key structuring forces shaping parasite communities. Along with the findings from other studies on fish–parasite networks, they shed further light on the ecology and evolution of host–parasite associations.

## Methods

### Network data compilation

A topic search of the Web of Science database was conducted in December 2021 using the search string: fish* AND (parasit* OR endoparasit* OR ectoparasit* OR helminth*) AND (network*). The 211 publications retrieved by the search were checked individually to identify those that provided a dataset, either available as Supplementary material or from a public repository, on fish–parasite bipartite interaction networks. We considered only networks involving metazoan parasites, from either freshwater or marine systems. If a few non-metazoan parasites were included in a network, we excluded them but still retained the network for further analysis. Some publications provided data from multiple networks, whereas some networks were re-used in more than 1 publication; we used each unique network only once in our analysis. When different versions of the same network were available, we only used the most complete one, i.e. the one with the most host and parasite species included. Here, we define a network as a set of fish and parasite species that co-occur in space and that can therefore potentially encounter each other and physically interact. In other words, we consider only local communities (e.g. a lake, a river stretch, a defined coastal area) as networks, and excluded all studies that assembled networks from continent-wide occurrence data (e.g. Braga *et al.*, 2014; Cruz-Laufer *et al.*, 2021). Finally, all networks were unweighed (providing only presence or absence of each parasite species on each host species), and treated as such in analyses. In the end, our set of networks was almost the same as that compiled by Bellay *et al.* (2013, 2015).

For each network, we recorded the following whole-network properties: the number of host species, the number of parasite species, the number of host–parasite links and the latitude of the network locality (estimated using Google Maps if not given in the original study). Additionally, each parasite species in each network was classified by (i) mode of infection, either *via* trophic transmission or by contact with external surfaces (whether or not tissue penetration ensued), and by (ii) higher taxon, i.e. myxozoans, hirudineans, molluscs, branchiurans, isopods, copepods, monogeneans, larval trematodes, adult trematodes, cestodes, nematodes and acanthocephalans. Larval and adult trematodes were classified separately because of their different mode of transmission: larval cercariae attach to and penetrate fish skin to settle as metacercariae within fish tissue, whereas adult trematodes are acquired by ingestion of infected intermediate hosts.

### Network analyses

All analyses were carried out in R (R Core Team, 2022). For each network, using the package *bipartite* v. 2.16 (Dormann *et al.*, 2008), we computed connectance, nestedness and modularity. Connectance and nestedness were calculated using the *networklevel* function, whereas modularity was calculated using the *computeModules* function. Connectance can vary between 0 and 1 (when all possible links are realized). Nestedness was measured as Weighted Nestedness based on Overlap and Decreasing Fill, or WNODF (Almeida-Neto and Ulrich, 2011); values can range between 0 (not nested) and 100 (fully nested). Modularity was estimated using the *Q* measure proposed by Newman and Girvan (2004), which ranges from 0 (prevalent links among modules) to 1 (most links within modules). Since WNODF and *Q* values are influenced by network size (total number of host and parasite species), they cannot readily be compared among networks. Instead of attempting to standardize them, we simply included network size as a predictor in the analyses (see below), to directly control for its influence on the estimates of nestedness and modularity.

We confirmed that the 3 network properties are not fully independent and covary with each other using pairwise Pearson's correlation coefficients: connectance *vs* nestedness: *r* = 0.898; connectance *vs* modularity; *r* = −0.608; nestedness *vs* modularity: *r* = −0.510 (all *P* < 0.005). We then tested for latitudinal gradients in these network properties, while controlling for variation in network size. For this, we used generalized linear models, 1 for each of the 3 network properties as response variables, with both latitude (absolute value, regardless of north or south) of the network and its size (sum of host and parasite species) as predictors. For these analyses, a gamma distribution was fitted to the connectance and nestedness data, whereas a Gaussian distribution was fitted to the modularity data.

### Species-level analyses

Again using the package *bipartite*, we used the *species level* function to calculate both the betweenness centrality and the closeness centrality (defined in the Introduction) values of each parasite species in each network. Centrality measures are widely used to assess species importance to the structure of host–parasite networks. They identify the species that maintain the cohesion of the network by connecting or linking host species (Martín González *et al.*, 2010). Parasite species with a disproportionate number of host interactions or that connect otherwise unconnected groups of parasite species into the network have higher centrality values and represent connectors; in contrast, parasite species with little or no importance for the cohesiveness of the network have values close to or equal to 0, and represent peripheral parasites (Martín González *et al.*, 2010).

The 2 main predictors, i.e. parasite higher taxon and mode of transmission, that we are investigating are confounded, because for most higher parasite taxa the mode of transmission is the same for all species. Therefore, we tested their effects in separate analyses. With these 2 predictors tested separately on 2 response variables (betweenness centrality and closeness centrality), we therefore ran 4 generalized linear mixed models (GLMMs) fitted with a gamma distribution using the *lme4* package (Bates *et al.*, 2015). The predictor ‘higher parasite taxon’ had multiple levels (12 higher taxa), whereas the predictor ‘transmission mode’ only had 2 levels (trophic transmission or skin contact). In the analyses of closeness centrality, the relatively few 0 values were replaced with 0.000001, which allowed using a gamma distribution without biasing the results. In contrast, for the analyses of betweenness centrality, we excluded all parasite species with a value of 0, which corresponds to species interacting with a single host species in their network, because there were too many of them. In all GLMMs, we also (i) included the number of host species used by a parasite as an additional predictor, to account for the influence of host specificity on the estimates of centrality, and (ii) included network identity as a random factor, to account for the non-independence of parasite species from the same network.

## Results

**Fig. 1.**
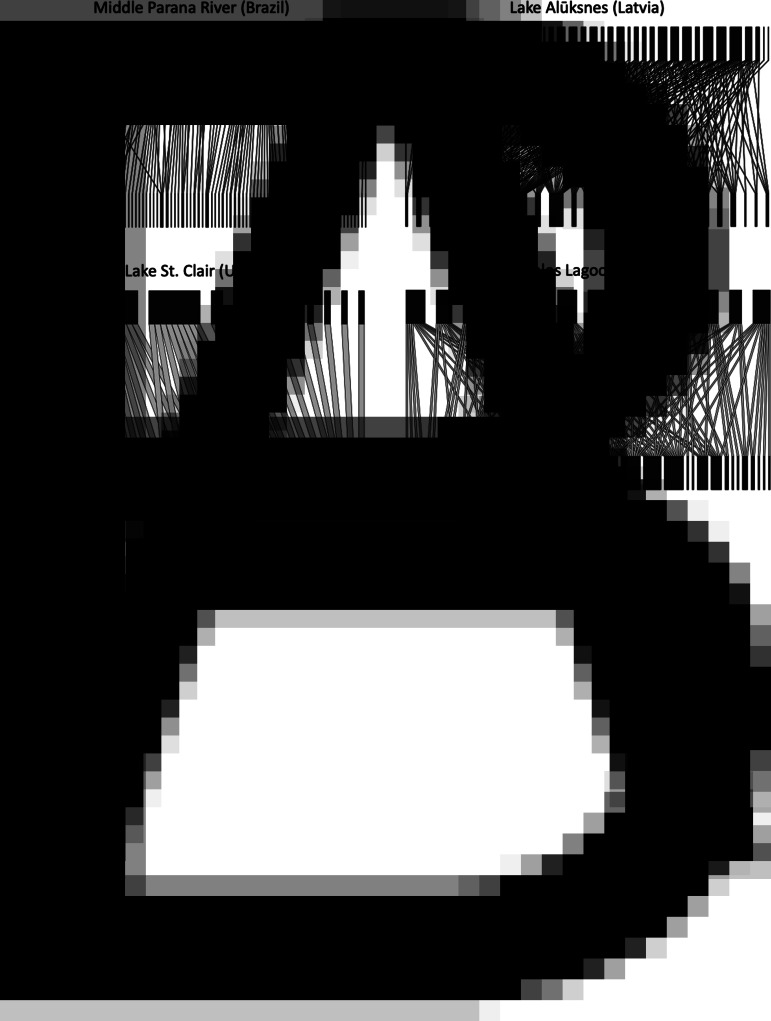
Examples of fish–parasite bipartite networks, with the fish hosts (top) and the parasites (bottom) represented by black rectangles, and the links between them indicated by connecting lines. (A) Two networks with similar numbers of fish hosts but very different connectance (Middle Parana River = 54 fish species, low connectance; Lake Alūksnes = 48 fish species, high connectance). (B) Two networks with identical numbers of fish hosts but very different nestedness (Lake St. Clair = 13 fish species, low nestedness; Tres Palos Lagoon = 13 fish species, high nestedness).

In total, we included data from 31 fish–parasite interaction networks (Table 1). These spanned almost 90° of latitude from north to south (most are from the Northern Hemisphere), and comprised from 6 to 91 fish species, from 14 to 420 parasite species and from 31 to 1085 host–parasite interaction links. Across networks, the numbers of host and parasite species covaried strongly (Pearson's correlation coefficient: *r* = 0.820, *N* = 31, *P* < 0.0001). The 31 networks also showed much variation in structure based on their basic properties (Fig. 1). Both the network-level and parasite species-level datasets are available in Supplementary material.

**Table 1.** Fish–parasite interaction networks considered here, along with their basic properties

Network locality	Country	Latitude	No. of fish species	No. of parasite species	No. of links	Reference
Lake Erie	USA and Canada	42.16	91	284	1085	Bellay *et al.* (2015)
Gulf of Tonkin	Vietnam	19.82	80	214	523	Bellay *et al.* (2015)
Lake Huron	USA and Canada	45.07	79	282	961	Bellay *et al.* (2013)
Upper Parana River basin	Brazil	−22.75	72	323	510	Lima *et al.* (2012)
Floodplain Upper Parana River	Brazil	−22.72	65	309	472	Bellay *et al.* (2015)
Lake Ontario	USA and Canada	43.73	61	246	586	Bellay *et al.* (2015)
Coastal Waters of Rio de Janeiro	Brazil	−22	59	420	709	Bellay *et al.* (2015)
Middle Parana River	Brazil	−29.82	54	93	146	Bellay *et al.* (2015)
Gulf of Riga	Latvia	57.11	52	95	469	Bellay *et al.* (2013)
Mekong River Delta	Vietnam	10.28	52	126	280	Bellay *et al.* (2013)
Lake Alūksnes	Latvia	57.45	48	22	247	Bellay *et al.* (2013)
Lake Raznas	Latvia	56.34	48	87	490	Bellay *et al.* (2013)
Lake Michigan	USA	43.59	45	108	230	Bellay *et al.* (2013)
Bay of Bengal	Bangladesh	21.11	37	49	77	Bellay *et al.* (2013)
Lake Superior	USA and Canada	47.83	36	165	379	Bellay *et al.* (2013)
Lake of the woods	Canada	49.07	31	138	362	Bellay *et al.* (2013)
St. Mary's River	USA and Canada	46.26	26	44	99	Bellay *et al.* (2013)
Guandu River	Brazil	−22.8	22	85	141	Bellay *et al.* (2015)
Parsnip River	Canada	54.44	17	53	158	Bellay *et al.* (2015)
McGregor River	Canada	54.3	14	51	114	Bellay *et al.* (2015)
Lake St. Clair	USA	42.44	13	31	40	Bellay *et al.* (2013)
Tres Palos Lagoon	Mexico	16.8	13	40	132	Bellay *et al.* (2015)
Hidvégi Lake	Hungary	46.63	12	34	51	Bellay *et al.* (2013)
Little Colorado River	USA	36.18	11	20	50	Bellay *et al.* (2015)
Cold Lake	Canada	54.5	10	37	91	Bellay *et al.* (2015)
Coyuca Lagoon	Mexico	16.95	10	34	104	Bellay *et al.* (2015)
Łebsko Lagoon	Poland	54.72	8	14	33	Bellay *et al.* (2013)
Zarivar Lake	Iran	35.54	8	20	31	Bellay *et al.* (2013)
Aishihik Lake	Canada	61.47	7	27	78	Bellay *et al.* (2015)
Devils Lake	USA	43.42	6	21	31	Bellay *et al.* (2013)
Smallwood Reservoir	Canada	54.12	6	24	53	Bellay *et al.* (2015)

The networks are listed in descending order based on the number of fish species they include. The reference given is the one from which data matrices were obtained, and not necessarily the one where the data were first presented.

### Network-level patterns

**Fig. 2.**
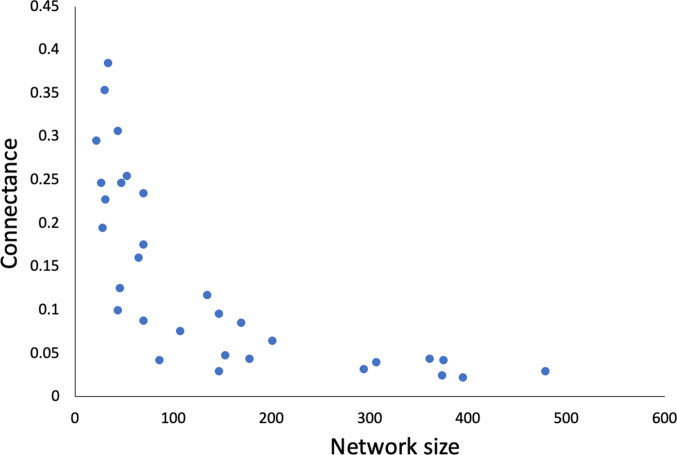
Connectance of fish–parasite interaction networks as a function of their size (sum of the numbers of host and parasite species).

**Fig. 3.**
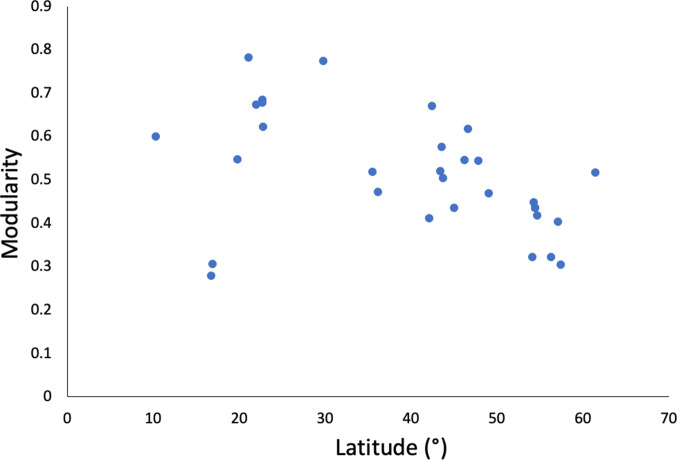
Modularity value of fish–parasite interaction networks as a function of their latitude (regardless of north or south).

Across networks, values of both connectance and nestedness decreased with increasing network size, whereas modularity was independent of network size (Table 2). Therefore, larger interaction networks consisting of more species of fish and parasites have disproportionally fewer host–parasite links (Fig. 2) and tend to be less nested. We also found that of the 3 network properties considered here, only modularity covaried significantly with latitude (Table 2). Modularity values decreased with increasing latitude (Fig. 3), indicating that fish–parasite networks at higher latitudes tend to be less modular, while those closer to the equator are more modular, with most links occurring within distinct subsets of interacting species. The latitudinal gradient would be even clearer if 2 data points, corresponding to 2 Mexican lagoons (in lower left portion of Fig. 3), were excluded.

**Table 2.** Results of generalized linear models testing the effects of latitude and network size (sum of host and parasite species) on 3 key network properties: connectance, nestedness and modularity

Response	Predictor	Estimate	Standard error	*t*-value	*P*
Connectance	Intercept	−1.826	0.299	6.092	<0.001
	Network size	−0.005	0.001	7.888	<0.001
	Latitude	0.009	0.006	1.510	0.142
Nestedness	Intercept	3.315	0.438	7.573	<0.001
	Network size	−0.005	0.001	5.518	<0.001
	Latitude	−0.001	0.009	0.128	0.899
Modularity	Intercept	0.616	0.081	7.582	<0.001
	Network size	0.000	0.001	1.007	0.322
	Latitude	−0.003	0.002	2.062	0.048

### Species-level properties

**Fig. 4.**
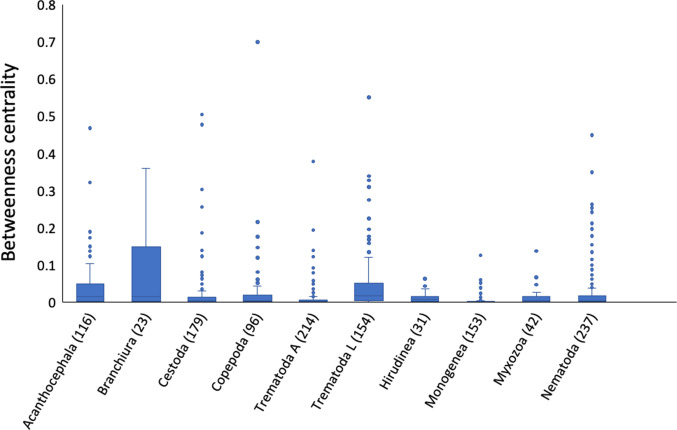
Betweenness centrality values (median and interquartile range) of parasite species within interaction networks with fish hosts, shown separately by parasite higher taxon. Only taxa with more than 20 species are shown; species with a centrality value of 0 are excluded. The number of species included is given in parentheses after each taxon's name.

Treating each species from a given network as unique (i.e. not accounting for the same species actually occurring in more than 1 network), the analysis comprised 3488 parasite species. The generalized linear mixed models found no evidence that a parasite's mode of transmission (trophic transmission *vs* skin contact) had any impact on its role within the interaction network as measured by either closeness or betweenness centrality (Tables 3 and 4). The number of host species used by a parasite, i.e. its host specificity, emerged as the main determinant of its centrality within the network. However, independently of the effect of the number of hosts used, there were differences in betweenness centrality among higher taxa of parasites (Table 4). Branchiurans, acanthocephalans and larval trematodes generally had higher centrality values than other parasite taxa (Fig. 4). In the analyses of betweenness centrality, species with a centrality value of 0, corresponding to species interacting with a single host species, were excluded. Since the proportion of species with a centrality value of 0 was lower among branchiurans, acanthocephalans and larval trematodes than among other parasite taxa (46 *vs* 67%), the greater betweenness centrality of species in these 3 taxa is actually even more pronounced than suggested by Fig. 4.

**Table 3.** Analysis of variance table summarizing the results of the GLMMs with closeness centrality of parasite species within interaction networks as the response variable, showing the effects of the main predictors

Predictor	*χ*^2^	Degrees of freedom	*P* value
Effect of parasite taxon			
Parasite taxon	8.99	12	0.960
No. of host species used by the parasite	35.15	1	<0.0001
Effect of mode of transmission			
Transmission mode	0.04	12	0.847
No. of host species used by the parasite	42.80	1	<0.0001

**Table 4.** Analysis of variance table summarizing the results of the GLMMs with betweenness centrality of parasite species within interaction networks as the response variable, showing the effects of the main predictors

Predictor	*χ*^2^	Degrees of freedom	*P* value
Effect of parasite taxon			
Parasite taxon	173.31	12	<0.0001
No. of host species used by the parasite	314.50	1	<0.0001
Effect of mode of transmission			
Transmission mode	0.01	12	0.935
No. of host species used by the parasite	373.63	1	<0.0001

Species with a centrality value of 0 were excluded from the analyses.

## Discussion

Treating communities of hosts and their parasites as interacting networks provides a comprehensive view of community structure, with the same analytical framework capable of addressing questions ranging from the species level to the assemblage level (Poulin, 2010; Delmas *et al.*, 2019; Runghen *et al.*, 2021). Network analysis is proving a powerful tool to identify constraints and drivers of community assembly, as well as predict the responses of communities to perturbations (Poisot *et al.*, 2016). Here we used this approach to determine whether basic properties of fish–parasite interaction networks show a latitudinal gradient after controlling for network size, and whether the position of individual parasite species within networks is associated with their taxonomy or transmission mode. Our analysis illustrates the usefulness of network analysis for investigations of host–parasite community structure and its determinants.

At the whole-network level, some of our findings support earlier ones. Across networks, the numbers of host and parasite species were strongly correlated, a pattern almost universally observed in comparisons across communities (Kamiya *et al.*, 2014). We also found that connectance decreases exponentially with increasing network size (number of species in the network), a result already found previously by Bellay *et al.* (2013) on almost the same network dataset, as well as by other comparative studies of host–parasite networks (e.g. Mouillot *et al.*, 2008). Furthermore, as observed in most types of bipartite interaction networks in ecology (Delmas *et al.*, 2019), we found that connectance correlates positively with nestedness and negatively with modularity.

More interestingly, we found that after accounting for variation in network size, fish–parasite networks at higher latitudes were only weakly modular, while tropical ones were more distinctly modular. We observed no latitudinal trend for either connectance or nestedness values. There have been very few attempts to find latitudinal gradients in the properties of antagonistic interaction networks (e.g. Guilhaumon *et al.*, 2012), and to our knowledge, this is the first to report a latitudinal gradient in modularity. The modularity *vs* latitude relationship we observed is even stronger when 2 data points are excluded (see Fig. 3, lower left portion). These correspond to 2 lagoons in Mexico (Violante-González and Aguirre-Macedo, 2007; Violante-González *et al.*, 2007), the only 2 networks among the ones considered here composed of a mixture of freshwater and marine species. It is possible that the disparate origins of hosts and parasites in these lagoons created incompatibilities (e.g. inability of freshwater parasites to infect marine hosts) that weakened the network's modularity. Alternatively, the low host specificity of many species of larval trematodes in these lagoons may have also contributed to reducing modularity. Strongly modular networks, in which most host–parasite links occur within distinct subsets of species, are essentially compartmentalized. Strong modularity is thought to promote community stability, because the impacts of perturbations such as extinctions are contained within a module and unlikely to spread to the rest of the community (Stouffer and Bascompte, 2011). Strong modularity at low latitude may be the result of host–parasite associations being more likely to form small coevolutionary units, i.e. small groups of host and parasite species that evolve in tandem more or less independently of the rest of the community, in these species-rich environments (Delmas *et al.*, 2019). Therefore, not only are tropical host–parasite networks generally characterized by higher species richness (Willig *et al.*, 2003) and greater consumer specialization (Vázquez and Stevens, 2004; Krasnov *et al.*, 2008), but also their more distinct modular structure may influence their ecological persistence and the evolutionary trajectory of their species.

At the species level, generalist parasites, that is, those that use many host species, not surprisingly emerged as occupying central positions within networks. When accounting for the influence of the number of host species used, transmission mode had no effect on species' centrality measures. Instead of splitting parasites based on their mode of transmission, Bellay *et al.* (2013) divided them based on whether they used a fish as an intermediate host (larval parasite) or as a definitive host (adult parasite), whereas Bellay *et al.* (2015) divided them based on their site of infection (ecto *vs* endoparasites). Bellay *et al.* (2013) found that parasite species occurring at larval stages within a network are involved in more links with hosts and in more among-module links, indicating greater centrality. In line with those findings, we found that larval trematodes have greater betweenness centrality values than adult trematodes. Independently of how many host species they use, we found that certain taxonomic groups, namely branchiurans, acanthocephalans and larval trematodes, have higher betweenness centrality values than other taxa of parasites. Since species with high betweenness centrality values are generally module connectors that contribute to whole-network cohesion (Delmas *et al.*, 2019), this may reflect the ability of parasites in those 3 groups to infect host species that are phylogenetically distant. There is indeed evidence that generalist species in these 3 groups infect not only many host species, but distantly related hosts (Poulin and Mouillot, 2003; Poly, 2008), resulting in their more influential role in shaping network structure. The role or position of a species in a network is not determined solely by its taxonomic affiliation, however; a large-scale analysis of helminth–vertebrate interactions indicates that a parasite species' role is not conserved across networks (Dallas and Jordano, 2021), suggesting that the local community context also influences the patterns of species interactions.

Several recent studies have highlighted the usefulness of network analysis to explore various aspects of fish–parasite community interactions and evolution. For example, a network approach can be used to assess the vulnerability of different types of parasites to local host extinction (Bellay *et al.*, 2020), or identify the host species most essential for the maintenance of local parasite diversity (Dallas and Cornelius, 2015). Network analysis can also provide insights into the impact of invasive fish species on host–parasite community structure (Llopis-Belenguer *et al.*, 2020). As with all approaches, however, the reliability of the results obtained through network analysis depends on the quality of the data. Most fish–parasite network datasets available at present have been assembled based exclusively on the morphological identification of parasite species. Cryptic species are frequently found when gene markers are used to distinguish among morphologically similar helminths (Pérez-Ponce de León and Poulin, 2018). As a consequence, both network-level and species-level metrics are likely to change when host specificity and host–parasite associations are re-assessed with molecular data (Poulin and Keeney, 2008). Also, most existing fish–parasite networks probably have many ‘missing links’, i.e. host–parasite associations not yet observed and thus not included in the network dataset. Solutions to this problem exist (e.g. Farrell *et al.*, 2022) but are yet to be widely implemented. Nevertheless, considering host–parasite communities as bipartite interaction networks remains the most holistic approach currently available to tackle not only unresolved questions about the structure of particular communities (Runghen *et al.*, 2021), but also to identify the main drivers of variation in key properties across different communities (Pellissier *et al.*, 2018; Xing and Fayle, 2021). The lack of universal rules that has long impeded progress in parasite community ecology (Poulin, 2007) may be over, with network analysis increasingly uncovering general and predictable patterns in how host–parasite associations are organized across within and among communities.

## Data Availability

All network data files used in this study are available in the Supplementary material.

## Supplementary material

For supplementary material accompanying this paper visit https://doi.org/10.1017/S0031182022000944. click here to view supplementary material
